# Why Good Employees Do Bad Things: The Link between Pro-Environmental Behavior and Workplace Deviance

**DOI:** 10.3390/ijerph192215284

**Published:** 2022-11-18

**Authors:** Zhenglin Zhang, Haiqing Shi, Taiwen Feng

**Affiliations:** 1 School of Management, Xi’an Polytechnic University, Xi’an 710043, China; 2 School of Management, Harbin Institute of Technology, Harbin 150001, China; 3 School of Economics & Management, Harbin Institute of Technology (Weihai), Weihai 264209, China

**Keywords:** public-sphere pro-environmental behavior, private-sphere pro-environmental behavior, workplace deviance, psychological entitlement, rationalization of workplace deviance

## Abstract

Despite the significance of pro-environmental behavior (PEB) in the workplace, most of the existing studies have neglected its negative work outcomes. Drawing upon moral licensing theory and cognitive dissonance theory, we construct a conceptual model of the influence mechanism of employees’ PEB (i.e., public-sphere PEB, private-sphere PEB) on workplace deviance through psychological entitlement, and the moderating effect of rationalization of workplace deviance on the relationship between psychological entitlement and workplace deviance. Using two-stage survey data from 216 employees in China, we performed hierarchical regression analysis and structural equation modeling method to test our hypotheses. Our findings reveal that public-sphere PEB positively affects psychological entitlement, while private-sphere PEB negatively affects psychological entitlement. Psychological entitlement further positively affects workplace deviance. In addition, rationalization of workplace deviance strengthens the positive impact of psychological entitlement on workplace deviance. This study offers novel insights into the dark side of PEB literature by exploring the PEB–workplace deviance relationship. This study also contributes to managerial implications of how PEB leads to workplace deviance and how to address this issue.

## 1. Introduction

With the crisis of energy and the environment, environmental sustainability has been a crucial way for firms to balance the paradox between economic growth and environmental protection [1,2]. To solve environmental issues and gain a competitive edge, the focal firm needs to find an effective way to distinguish itself from other firms [3]. There is wide consensus that employees’ pro-environmental behavior (PEB) is one of the most successful ways to promote the sustainable development of firms [4,5,6,7,8]. Employees’ PEB refers to the proactive environmentally friendly behavior of employees in the workplace, which may be deemed as an “extra role” behavior [5,6]. Extant research has generally believed that employees’ PEB is a positive behavior, such as saving resources, reducing work costs and ensuring a competitive advantage for teams and organizations [9,10]. Because of the positive work outcomes of PEB, most of the current research focuses on how to encourage PEBs [5,6,11,12].

Although the positive side of PEB has been well understood, our knowledge of its potential drawbacks is still limited. Notably, a few studies have shown that PEB may bring adverse impacts. For example, some scholars have revealed that an individual’s PEB may lead to a negative spillover effect in some cases [13,14,15]. Even so, we know little about the negative work outcomes of employees’ PEB. In this study, we suggest that employees with PEB may engage in deviant behavior in the workplace. Specifically, employees’ PEB is not taken into account in KPIs to compensate them for their devotions in a commercial firm that emphasizes profit [16]. Employees may rethink whether the PEB is worth the cost. When the compensation for PEB is lower than the expected return, employees may engage in workplace deviance [17]. Furthermore, due to limited time- and energy resources, too much engagement in PEB may reduce employees’ in-role performance, which also leads to workplace deviance. Although workplace deviance has become a common problem in business practice, few studies have linked PEB to workplace deviance, and the underlying influence mechanism also remains unclear.

To bridge this research gap, drawing on moral licensing theory, we argue that employees’ PEB may lead to workplace deviance through psychological entitlement. From the perspective of moral licensing theory, employees’ PEB is typically viewed as a morally laudable behavior, which may induce their inflated perceptions and further trigger high-level psychological entitlement, which is similar to a “licensing effect” [13]. In turn, employees’ high-level psychological entitlement makes them feel that they deserve better treatment than their peers. Hence, when such higher expectations and skewed notions are not met, they may engage in workplace deviance to offset these unmet needs and seek psychological equivalence [18,19].

In addition, why do employees with PEB in the same workplace differ in their subsequent behaviors? We deem that the individual characteristics of employees may affect their understanding and perception of PEB, and then influence subsequent workplace deviance. In particular, we suggest that employees’ psychological entitlement triggered by their PEB and high-level rationalization of workplace deviance will interact to trigger subsequent workplace deviance. When engaging in workplace deviance, employees may feel anxious and uneasy due to violating the firm’s working rules and norms [20,21]. According to cognitive dissonance theory, rationalization of workplace deviance may help employees justify and make excuses for their workplace deviance resulting from psychological entitlement, and reduce employees’ anxiety and guilt to a certain extent. Thus, high-level rationalization of workplace deviance strengthens the link between psychological entitlement and workplace deviance.

In this study, we apply moral licensing theory and cognitive dissonance theory to reveal when and how employees’ PEB can trigger workplace deviance. The objectives of this study are to: (1) promote the understanding of the links among PEB, psychological entitlement, and workplace deviance and (2) explore how rationalization of workplace deviance prompts employees with psychological entitlement to engage in workplace deviance. Our results indicate that employees’ public-sphere PEB positively affects psychological entitlement, while private-sphere PEB negatively affects psychological entitlement. In turn, psychological entitlement positively affects workplace deviance. Finally, rationalization of workplace deviance strengthens the positive impact of psychological entitlement on workplace deviance.

The structure of our research is organized as follows. In Section 2, we review the literature and propose hypotheses. Section 3 introduces the research methods. In Section 4, we test the hypothesis and conduct a robust check. Section 5 discusses the results, and provides theoretical and managerial implications. The final section reaches conclusions and provides several limitations that leave room for future research.

## 2. Literature Review and Hypotheses

### 2.1. Moral Licensing Theory

**Figure 1:**
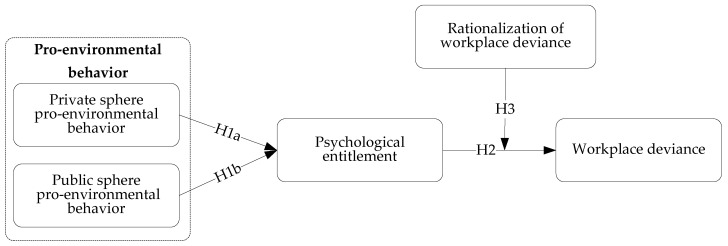
Conceptual model.

Moral licensing theory suggests that engaging in morally praiseworthy and socially desirable behavior can license people to follow with unethical behavior [18,22]. Moral licensing theory is particularly helpful for our research, which is conducive to explaining employees’ subsequent behavior that is inconsistent with their prior actions [23,24]. However, previous studies on the moral licensing effect in the environmental action domain are limited [13]. Gholamzadehmir et al. [13] proposed that PEB may lead to moral licensing, while they only focused on PEB’s negative spillover effect and did not extend subsequent unethical behavior into other domains. To fill this research gap, based on moral licensing theory, we suggest that after engaging in PEB, employees tend to form a license of psychological entitlement, which further leads to subsequent workplace deviance. Drawing upon cognitive dissonance theory, we also argue that the rationalization of workplace deviance can moderate psychologically entitled employees’ workplace deviance. As shown in Figure 1, we next develop our hypotheses.

### 2.2. PEB and Workplace Deviance

PEB refers to behavior benefiting environmental protection, whose goal is to remain the sustainability of the natural and social environment [2,6,25]. Individual’s PEB has lots of benefits ranging from the household to workplace surroundings [15]. Actually, no matter the implementation of environmental protection projects or the management of daily business operations, every link of the focal firm cannot be separated from the participation of employees. If employees’ behavior is pro-environment, it will undoubtedly greatly benefit the environmentally sustainable development of the focal firm [9,26,27]. Specifically, employees’ PEB can conserve resources and energy for the focal firm, such as printing double-sided, reusing recyclable materials and using the stairs instead of the lift, etc., which further is conducive to reduce work costs and ease the burden of the focal firm [2,9]. In summary, employees’ PEB plays a direct and essential role in ensuring an organization’s longevity, success and competitive advantage [10].

However, although the positive side of PEB has been well understood, our knowledge of its negative side is still limited. Notably, some studies have shown that PEB may also bring a negative spillover effect [13]. That is, an individual’s single PEB will reduce other PEBs [14,15]. Apart from PEBs’ negative spillover effects, few studies have examined employees’ PEB negative impact in the workplace. To fill this research gap, we argue that employees’ PEB may lead to workplace deviance under certain conditions.

Workplace deviance refers to the spontaneous behavior of employees, which is a violation of the rules, policies or institutions of the firm, and threatens the benefits or the internal members of the firm [28]. For specific performances, such as decrease in work time or work efforts which result in low efficiency, poor work performance and relationships, low job satisfaction and idling in work to postpone scheduled work [28]. Specifically, employees’ PEB is not taken into account in KPIs to compensate them for their devotions in a commercial firm that emphasizes profit [16]. Employees may rethink whether the PEB is worth for their cost. When the compensation of PEB is lower than their expected return, employees may engage in workplace deviance [17]. Furthermore, due to limited time and energy resources, too much engagement in PEB may reduce employees’ in-role performance, which also leads to workplace deviance. However, even though PEB may engage in workplace deviance, its impact on workplace deviance lacks empirical support, and the underlying influence mechanism also remains unclear.

Suggested by moral licensing theory, the engagement of employees in moral behavior provides them a “license” that can engage in unethical behaviors in the future. Moral licensing theory may provide an opportunity to uncover the influences of PEB on workplace. Thus, we propose psychological entitlement, which represents the psychological state of moral license, as a linkage for uncovering details of the link between PEB and workplace deviance.

### 2.3. PEB, Psychological Entitlement and Workplace Deviance

#### 2.3.1. PEB and Psychological Entitlement

Employees’ PEB can be categorized into public-sphere PEB and private-sphere PEB, based on the different space divisions in the context of China [2]. Specifically, private-sphere PEB refers to the establishment of employee self-discipline-oriented PEB, the generation of which usually depends on pro-environment values and the transitive role of family-oriented PEB [2]. By contrast, public-sphere PEB refers to organized and interactive pro-environment activities that reflect the concern of employees and other organization members about environmental issues [2]. Some scholars indicated that PEB can trigger a moral licensing effect and a negative spillover effect [13]. Nevertheless, whether both employees’ private-sphere PEB and public-sphere PEB induce the moral licensing effect is unknown.

According to moral licensing theory, we argue that both private-sphere PEB and public-sphere PEB may trigger the moral licensing effect to further generate psychological entitlement, which is analogous to moral licensing [18]. Psychological entitlement refers to the sense of deserving more payback including time, money, leisure, respect or other resources, which dominates many human behaviors in the social exchange context [29,30]. Psychological entitlement can be seen as a state of mind and can be enabled by certain situational experiences [3,31] We thus deem that private-sphere PEB and public-sphere PEB may activate psychological entitlement in different pathways.

If there is a need to promote public-sphere PEB which is less representative of their own interests, employees often need to pay a high price (time, energy, money, etc.) [2]. Based on moral licensing theory, public-sphere PEB is generally regarded as a praiseworthy behavior; such a high cost makes employees feel that they deserve more resources and compensation than others, and this perception is psychological entitlement [32]. Therefore, we expect a positive effect of public-sphere PEB on psychological entitlement. Compared to public-sphere PEB, private-sphere PEB is often motivated spontaneously, which can be considered rare in the workplace [2]. According to moral licensing theory, employees who engage in private-sphere PEB may feel that they are unique and different, and have the right to obtain special treatment different from others. Such a sense of entitlement is usually manifested as psychological entitlement. Thus, we argue that private-sphere PEB is positively related to psychological entitlement. We posited the following two hypotheses:

**Hypothesis** **1** **(H1).** *(a) Private-sphere PEB and (b) public-sphere PEB relate positively to psychological entitlement*.

#### 2.3.2. Psychological Entitlement and Workplace Deviance

Psychological entitlement refers to an individual’s subjective belief that he/she deserves more than others, even if it is not commensurate with his/her actual abilities and efforts [33], which is similar to the moral licensing effect [18]. Based on moral licensing theory, employees’ psychological entitlement implies moral permission, and then employees may conduct behaviors that do not conform to the moral standards. Indeed, psychological entitlement is a common psychological phenomenon widely existing in the workplace, which is generally relevant to negative outcomes [18,34]. For instance, employees’ psychological entitlement can result in a variety of adverse reactions in attitudes and behaviors, among which is the threat of workplace deviance [18]. Therefore, we further propose that employees’ psychological entitlement can lead to workplace deviance.

Employees with high-level psychological entitlement generally have inflated expectations and tend to expand self-cognition and self-service attribution, and believe they deserve to be treated specially and more richly rewarded than others [19,35]. Nevertheless, these inflated expectations are not easily satisfied and there is a wide disparity between what employees expect and what they actually obtain [36]. Then, they may feel the psychological imbalance and use improper means to offset their unmet expectations and needs [18,19]. As a result, employees may engage in workplace deviance to seek psychological equivalence. Furthermore, employees with high-level psychological entitlement may truly believe that their workplace deviance is justified and reasonable, and will not be condemned by their conscience. They may see workplace deviance as a fair response to PEB although their PEB is highly exaggerated by psychological entitlement [19]. These findings lead to the following hypothesis:

**Hypothesis** **2** **(H2).** *Psychological entitlement is positively related to workplace deviance.*

### 2.4. The Moderating Effect of Rationalization of Workplace Deviance

Rationalization of behavior is the nature of people to act when they encounter a dilemma including moral and behavioral choice [37]. According to cognitive dissonance theory, people may try to minimize anxiety by pursuing tranquility of heart when facing confusion about a decision [38]. For instance, people can experience strong psychological conflict when they encounter an inconsistency of attitude and behavior. Facing the conflict of motivations and a need to regard the self as moral, people usually want to stay in a harmonious spiritual state and struggle to address the conflict. Some behaviors, such as unethical behaviors, have too much fascination that people mostly change the belief rather than the behaviors [20,21]. Therefore, the rationalization of behavior is particularly common where people try to seek a reasonable subjective persuasion to verify the rightness of the preceding decision. We further examine the moderating role of the rationalization of workplace deviance between psychological entitlement and workplace deviance.

As mentioned earlier, psychological entitlement can induce workplace deviance. Employees may feel anxious and uneasy due to workplace deviance that violates the working rules and norms of the focal firm [20,21]. From the perspective of cognitive dissonance theory, it is easy for employees to generate a reasonable explanation of the actual workplace deviance to balance their ambivalence. The rationalization of workplace deviance can largely reduce employees’ anxiety and guilt of workplace deviance resulting from psychological entitlement [39]. It also helps employees make excuses for their workplace deviance and rationalize their workplace deviance. In this view, employees believe that workplace deviance is a “natural” practice and a fair response to offset psychological entitlement, even though they bring losses to their organization and its members at a certain cost. Therefore, we argue that when the level of rationalization of workplace deviance is high, employees’ psychological entitlement makes it easier for them to engage in workplace deviance. We predict the following:

**Hypothesis** **3** **(H3).** *Rationalization of workplace deviance positively moderates the relationship between psychological entitlement and workplace deviance*.

## 3. Methods

### 3.1. Data Collection

The research topic of this study covers psychological entitlement and workplace deviance, which are sensitive issues that involve respondents’ privacy. In the context of China, respondents are often wary of being surveyed by strangers, and it is difficult to guarantee the quality of questionnaire completion [40]. Given that, we adopted convenience sampling to collect survey data from employees through self-administered questionnaires [41]. We contacted with five firms in Xi’an to distribute our questionnaires. The five firms cover distinct industries, including automotive, special equipment, pharmaceutical manufacturing, food processing and communication. Among them, two firms in the automotive industry and special equipment industry are state-owned, while the other three are privately-owned. In each firm, a human resource manager was identified to assist us in sending the questionnaire to full-time employees. We randomly selected 100 respondents in each firm according to a named list of employees. We then sent the questionnaires including information concerning the academic purpose and the assurance of confidentiality to respondents.

We conducted this survey in two stages to reduce the potential threat of common method variance (CMV), spaced approximately 2 weeks apart. In the first stage, we collected data comprising basic information about employees, PEB and psychological entitlement. In the second stage, we mainly included items related to workplace deviance and rationalization of workplace deviance. Of the 500 respondents who agreed to participate, a total of 227 employees completed the survey across two different time-points. Finally, after eliminating 11 invalid questionnaires that could not be matched, missed or were carelessly filled out, we received a total of 216 valid questionnaires. The valid response rate was 43.2% (216 out of 500). The average age of informants was 31.9 years (SD = 8.2). A total of 56.5% of the respondents were married, while 43.5% of them were unmarried or divorced. A total of 50.0% respondents were male, and an average experience in the firm of 9.2 years (SD = 9.2). The average annual income of respondents were 75,700 Chinese yuan (SD = 21.5). From the sample situation, the sample distribution is reasonable and has good representativeness.

### 3.2. Measures

In this study, the measurement instruments were all derived from mature scales, and we properly adapted the related scales to ensure the effectiveness in a Chinese context. We employed a pilot study with 30 employees, according to the feedback from which we modified the initial questionnaire. We adopted standard translation–retranslation procedures. The scales were assessed with a 7-point Likert scale ranging from 1 = ‘strongly disagree’ to 7 = ‘strongly agree’. The list of measurement items is shown in Appendix A.

PEB. In this study, we categorized PEBs into private-sphere PEB and public-sphere PEB, both of which were measured with a four-item scale adapted from Lu et al. [2] and Robertson and Barling [42]. Sample items of private-sphere PEB were “I print double sided whenever possible”, and “I maintain office equipment”. The Cronbach’s alpha for the scale was 0.882. Sample items of public-sphere PEB were “I support positive implementation of national and organizational environmental policies” and “I make suggestions about environmentally friendly practices to managers in an effort to increase my organization’s environmental performance”. The Cronbach’s alpha for the scale was 0.858.

Psychological entitlement. We measured psychological entitlement with a four-item scale adapted from Wetzel et al. [30] and Yam et al. [30]. Sample items were “I honestly feel I’m just more deserving than others in my firm”, and “I demand the best because I’m worth it”. The Cronbach’s alpha for the scale was 0.837.

Rationalization of workplace deviance. We measured rationalization of workplace deviance with a five-item scale adapted from Jamie et al. [43]. Sample items were “I generally engage in various types of workplace deviance in order to compete effectively”, and “Workplace deviance behavior is a normal part of work”. The Cronbach’s alpha for the scale was 0.795.

Workplace deviance. We measured workplace deviance with a six-item scale adapted from Bennett and Robinson [28] and Yam et al. [18]. Sample items were “I work on my personal matter instead of working for my firm”, and “I leave my work for someone else to finish”. The Cronbach’s alpha for the scale was 0.898.

Control variables. Previous studies have proposed that an individual’s workplace deviance may be affected by demographic variables, including gender, marital status, education, age, tenure and salary [44,45]. Therefore, this study controlled the influences of these variables. Gender was measured with a dummy variable by coding male as “1” and coding female as “0”. Marital status was also assessed with a dummy variable by coding married as “1” and coding unmarried or divorced as “0”. Education was measured by two dummy variables with below bachelor degree as the base. We also controlled age, tenure and salary using their natural logarithms.

### 3.3. Reliability and Validity

According to a suggestion proposed by Fornell and Larcker [46], we calculated Cronbach’s alpha and composite reliability (CR) to assess the reliability of constructs. The results shown in Table 1 indicated that all the five Cronbach’s alpha and CR values were greater than 0.75. Thus, reliability was good in this study.

In this research, we employed CFA to examine convergent validity and discriminant validity. The model fit indices were satisfactory (χ^2^ = 539.04, d.f. = 220, RMSEA = 0.078, CFI = 0.93, NNFI = 0.94, SRMR = 0.059). As shown in Table 1, all the factor loadings were significant, indicating good convergent validity. Furthermore, we calculated average variance extracted (AVE) values to evaluate convergent validity [47]. All the AVE values were above 0.50, which offered further support for good convergent validity.

To examine discriminant validity, this study compared the square root of the AVE value of each construct with the correlations between this construct and the other constructs. If the square root of the AVE value was greater than the correlations, discriminant validity would be ensured [48]. Table 2 presented the square root of AVE values and the inter-correlations of all the constructs. The square root of AVE value for each construct was greater than the correlations with the other constructs which indicated satisfactory discriminant validity.

### 3.4. Non-Response and Common Method Bias

Non-response bias and CMV are commonly raised when employing survey methodology [49]. With the help of human resource managers, we compared several key demographic attributes (e.g., age, working years and gender) between respondents and non-respondents. The insignificant *t*-statistics suggest that no significant differences between respondents and non-respondents. We also split the 216 respondents into two groups according to their returning dates. The early group contained 149 samples, and the late group included 67 samples. The *t*-tests and ANOVA reveal insignificant differences among the two groups in the demographic characteristics. Overall, our results confirmed that non-response bias was not serious.

To evaluate the potential influence of CMV, we conducted Harman’s one-factor test [47]. Five different factors with an eigenvalue above 1.0 emerged, suggesting that CMV is not serious in our research. To further examine CMV, we compared the confirmatory factor analysis (CFA) model with the model adding a method factor [50]. The results demonstrated that the model including a method factor marginally improved the model fit indices (∆NNFI = 0.02 and ∆CFI = 0.02), with the common method factor explaining 4.6% of the total variance. In addition, the factor loadings were still significant after adding a method factor, suggesting a robust CFA model. As a result, the possible influence of CMV was not serious.

## 4. Results

This study performed hierarchical multiple regression analysis to examine the research hypotheses. Table 3 shows the results of regression analysis. We mean-centered psychological entitlement and rationalization of workplace deviance before calculating the interaction term to reduce the threat of multi-collinearity [51]. Further, we computed variance inflation factors (VIFs) to evaluate the influence of multi-collinearity. Our results suggest that the VIFs are lower than 10 in all models, indicating multi-collinearity is not an issue.

### 4.1. Hypotheses Testing

To examine the impacts of private-sphere PEB and public-sphere PEB on psychological entitlement, we conducted a two-step regression approach. At the first step, we included the control variables in Model 1. Second, we added private-sphere PEB and public-sphere PEB in Model 2. Results in Model 2 reveal that private-sphere PEB negatively influences psychological entitlement (*β* = −0.177, *p* < 0.05), while public-sphere PEB positively influences psychological entitlement (*β* = 0.238, *p* < 0.01). Hence, H1a is not supported while H1b is supported.

To test the effect of psychological entitlement on workplace deviance, we included the control variables in Model 3, and then added psychological entitlement in Model 4. The results in Model 4 suggest that psychological entitlement has a positive and significant effect on workplace deviance (*β* = 0.365, *p* < 0.001). Thus, H2 is supported.

**Figure 2:**
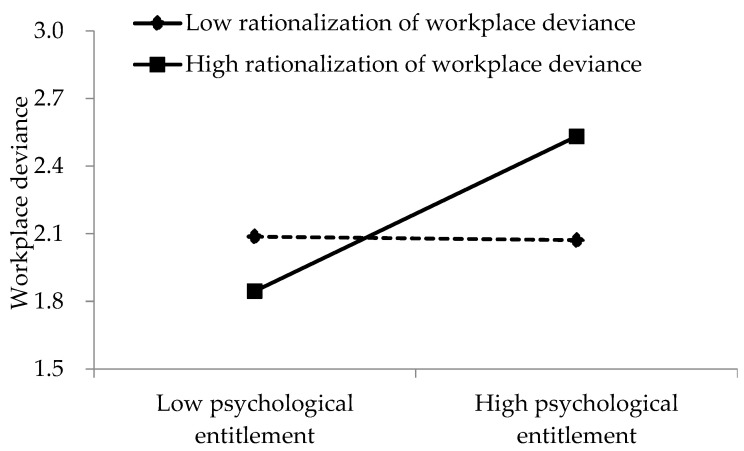
The moderating role of rationalization of workplace deviance.

Model 5 further added the moderating variable and the interaction term based on Models 3 and 4 to examine the moderating role of rationalization of workplace deviance. The results in Model 5 indicate that the interaction between rationalization of workplace deviance and psychological entitlement has a positive and significant impact on workplace deviance (*β* = 0.223, *p* < 0.001). Thus, H3 is supported. Moreover, we plotted this interaction to obtain more insight into the moderating effect [51]. Figure 2 indicates that when the level of rationalization of workplace deviance is high, the positive effect of psychological entitlement on workplace deviance is more significant, further supporting H3.

### 4.2. Robust Check

**Figure 3:**
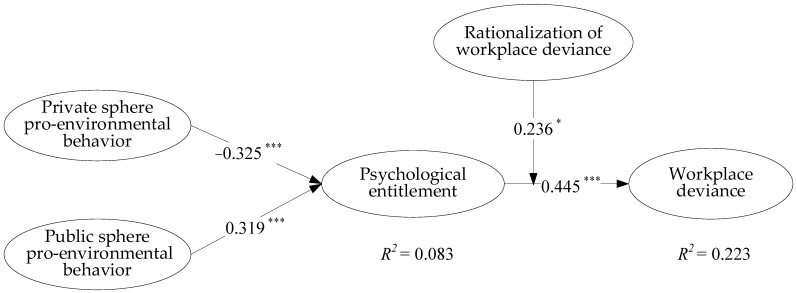
SEM results. Note: * *p* < 0.05, *** *p* < 0.001.

We employed the structural equation modeling (SEM) method with Mplus 8.0 software to test the robustness of the hypothesized relationships. As shown in Figure 3, the SEM results are basically consistent with the regression results, indicating the robustness of our model.

## 5. Discussion

### 5.1. Discussion of the Results

Although it is generally regarded that we should try our best to take feasible and effective actions to protect the environment, the dark side of PEB has been neglected. The purpose of our research was to develop and empirically examine a theoretical model that can explain how employees’ PEB leads to psychological entitlement and further engagement in workplace deviance.

In this research, we argue that both private-sphere PEB and public-sphere PEB relate positively to psychological entitlement. Psychological entitlement, in turn, relates positively to workplace deviance. Specifically, the results show that the public-sphere PEB positively affects psychological entitlement and psychological entitlement positively affects workplace deviance, as we hypothesized. This study is in line with several prior pieces of research that suggest employees’ positive behavior may also become a trigger of negative behaviors through psychological entitlement. For instance, Vincent and Kouchaki [3] proposed that employees with a creative identity may elicit psychological entitlement and further engage in unethical behaviors; Yam et al. [18] examined whether employees’ organizational citizenship behaviors may induce feelings of entitlement and further lead to interpersonal or organization-directed deviant behaviors; Loi et al. [52] confirmed the mediating effect of psychological entitlement between employee volunteering and workplace deviance.

Interestingly, contrary to our hypothesis, the results show that the private-sphere PEB negatively affects psychological entitlement. The finding is similar to recent research revealing that engagement in PEB does not always lead to moral licensing [53]. One possible explanation is that the employees engaging in private-sphere PEB focus on self-discipline and self-satisfaction and rarely generate a sense of psychological entitlement [34]. Another possibility is that employees with private-sphere PEB may arise from PEB pro-environmental values and rarely demand more resources and compensation, which might not come with a sense of psychological entitlement.

Furthermore, based on cognitive dissonance theory, we explain how rationalization of workplace deviance interacts with psychological entitlement to induce workplace deviance. We argue that under high-level rationalization of workplace deviance, employees are more likely to engage in workplace deviance driven by psychological entitlement. Our results provide empirical support for this argument, which indicates that when rationalization of workplace deviance is high, employees are more prone to workplace deviance if they feel psychologically entitled.

### 5.2. Theoretical Contributions

First, we enrich the literature about PEB by extending the discussion on its negative implications. Previous studies examined whether engagement in PEB may trigger adverse effects on subsequent PEB [54], while mostly ignoring the link between PEB and workplace deviance. This study helps address this imbalance by finding that investing more time and effort in public-sphere PEB may provide employees with a “license” and in turn increase the likelihood of engaging in workplace deviance. We also provide empirical support for the view that an employee can do both good and bad things, and that there may even be a positive link between the two seemingly opposite behaviors [3,8,52].

Second, based on moral licensing theory, we uncover the black box of employees’ PEB leading to workplace deviance from the perspective of psychological entitlement. Our results show that public-sphere PEB can motivate employees’ psychological entitlement, which can further influence their workplace deviance; meanwhile, private-sphere PEB negatively relates to psychological entitlement. The different influence pathways of public-sphere PEB and private-sphere PEB on psychological entitlement help resolve the inconsistent effects of PEB in prior research.

Finally, this study enriches the previous research on rationalization behavior. Drawing upon cognitive dissonance theory, our results indicate that employees’ rationalization of workplace deviance strengthens the positive effect of psychological entitlement on workplace deviance. This finding not only effectively reveals the boundary of PEB’s role in influencing workplace deviance, but also contributes to cognitive dissonance theory.

### 5.3. Managerial Implications

This study has several managerial implications for firms seeking to increase employees’ PEB. First, this study helps managers gain a richer understanding of private-sphere PEB and public-sphere PEB. Specifically, our findings reveal that public-sphere PEB positively affects psychological entitlement, while private-sphere PEB negatively affects psychological entitlement. Considering the potential moral hazards of public-sphere PEB, we suggest that managers should encourage employees’ PEB cautiously in practice instead of blindly requiring PEB, which may lead employees to trigger workplace deviance in order to gain more resources and compensation than others. Managers can consider reasonably increasing the rewards for PEB in the compensation system, so as to reduce to some extent employees’ perception of losses caused by PEB and the possibility of workplace deviance.

Second, this study found that psychological entitlement leads to workplace deviance. Psychological entitlement is an important factor that induces employees to engage in workplace deviance. Managers should pay attention to the psychological state of employees and create good psychological conditions for them. Managers need to take differentiated management measures and institutional flexibility for employees with different levels of psychological entitlement. At the same time, managers should pay more attention to those employees with a high-level psychological entitlement to avoid workplace deviance, such as increasing the frequency of communication with them and giving them humanistic care to the maximum extent in the workplace.

Finally, the rationalization of workplace deviance strengthens the positive effect of employees’ psychological entitlement on workplace deviance. In this view, employees argue that their workplace deviance is rationalized. Managers should consider thorough training, ethical policies and strong construction of the firm’s culture as ways for employees to recognize the serious consequences of workplace deviance. Compared to the benefits of PEB, workplace deviance leads to a bigger loss and allows firms to do more harm than good. Furthermore, measures can be taken to properly supervise workplace deviance, and let employees supervise and help each other, so as to reduce the probability of participating in workplace deviance.

## 6. Conclusions and Implications

This study offers new insights into the negative consequences of employees’ PEB. Our objective in this research is to advance the understanding of the relationships between the PEB, psychological entitlement and workplace deviance. Specifically, employees’ public-sphere PEB positively affects psychological entitlement, while private-sphere PEB negatively affects psychological entitlement. Further, psychological entitlement can indeed lead employees to engage in workplace deviance. What is more, the findings show that the positive impact of psychological entitlement on workplace deviance will become stronger as the level of rationalization of workplace deviance increases.

This research enriches the existing literature on PEB and deepens our understanding of PEB’s negative outcomes. However, there are still some limitations left for future research. First, we only explore the moderating role of rationalization of workplace deviance. Future research that enlarges on more moderators such as environmental identification or the organizational green atmosphere may provide a richer understanding of the consequences of PEB. Second, we used a self-assessment method to collect data. Although common method bias is not serious in our research, due to the sensitivity of research ethics, the self-assessment method is difficult to avoid the influence of social approval tendency on research results [55,56]. Longitudinal studies from multiple sources and multiple time points should be adopted in the future research. Finally, our data were mainly confined to five firms in Xi’an, China, which limits the generalizability of our results. Although respondents came from various industries, they may not be representative of the broader working population in China. Future research should consider enlarging the sample size as much as possible to provide geographic diversity and the representative of the sample.

## Figures and Tables

**Table 1:** CFA results.

Constructs	Item Code	Factor Loading	Cronbach’s Alpha	CR	AVE
Private-sphere PEB	PRPE1	0.82	0.882	0.890	0.670
	PRPE2	0.89			
	PRPE3	0.87			
	PRPE4	0.68			
Public-sphere PEB	PUPE1	0.86	0.858	0.867	0.626
	PUPE2	0.84			
	PUPE3	0.86			
	PUPE4	0.57			
Psychological entitlement	PE1	0.76	0.837	0.876	0.649
	PE2	0.53			
	PE3	0.96			
	PE4	0.90			
Rationalization of workplace deviance	RWD1	0.77	0.795	0.833	0.503
	RWD2	0.75			
	RWD3	0.61			
	RWD4	0.60			
	RWD5	0.79			
Workplace deviance	WD1	0.64	0.898	0.904	0.563
	WD2	0.76			
	WD3	0.85			
	WD4	0.84			
	WD5	0.89			
	WD6	0.70			

**Table 2:** Mean, standard deviations and correlations of the constructs.

Variables	Mean	S.D.	1	2	3	4	5	6	7	8	9	10	11	12
1. Gender	0.500	0.501	-											
2. Marital status	0.565	0.497	−0.093	-										
3. Dummy of education 1	0.583	0.494	0.075	−0.325 ***	-									
4. Dummy of education 2	0.088	0.284	0.082	0.108	−0.367 ***	-								
5. Age	3.430	0.244	−0.094	0.686 ***	−0.335 ***	0.103	-							
6. Tenure	1.854	0.974	−0.038	0.659 ***	−0.389 ***	0.102	0.775 ***	-						
7. Salary	1.991	0.249	0.241 ***	0.132	−0.078	0.380 ***	0.074	0.053	-					
8. Private-sphere PEB	5.417	1.151	−0.054	0.213 **	0.016	−0.020	0.261 ***	0.196 **	−0.106	0.819				
9. Public-sphere PEB	4.943	1.161	−0.051	0.241 ***	−0.137 *	−0.140 *	0.283 ***	0.193 **	−0.039	0.534 ***	0.791			
10. Psychological entitlement	3.188	1.555	0.082	0.087	−0.089	−0.159 *	0.005	−0.045	0.100	−0.073	0.191 **	0.806		
11. Rationalization of workplace deviance	3.615	1.254	0.150 *	−0.064	−0.017	−0.043	−0.030	−0.071	0.085	−0.194 ***	−0.007	0.229 ***	0.709	
12. Workplace deviance	2.302	1.032	0.157 *	−0.107	−0.076	−0.051	−0.092	−0.136 *	0.063	−0.118	0.038	0.391 ***	0.478 ***	0.750

Note: * *p* < 0.05; ** *p* < 0.01; *** *p* < 0.001 (two-tailed); square root of average variance extracted is on the diagonal.

**Table 3:** The results of regression analysis.

Variables	Psychological Entitlement		Workplace Deviance		
	Model 1	Model 2	Model 3	Model 4	Model 5
Control variables					
Gender	0.094	0.092	0.159 *	0.125	0.070
Marital status	0.176	0.169	−0.063	−0.127	−0.091
Dummy of education 1	−0.224 **	−0.167 *	−0.220 **	−0.138	−0.088
Dummy of education 2	−0.308 ***	−0.247 **	−0.154 *	−0.042	−0.007
Age	0.022	−0.003	0.071	0.063	−0.024
Tenure	−0.239 *	−0.208	−0.218	−0.131	−0.048
Salary	0.165 *	0.138	0.081	0.021	0.003
Independent variables					
Private-sphere PEB		−0.177 *			
Public-sphere PEB		0.238 **			
Mediator					
Psychological entitlement (PE)				0.365 ***	0.325 ***
Moderator					
Rationalization of workplace deviance (RWD)					0.340 ***
Interaction					
PE × RWD					0.223 ***
R2	0.119	0.157	0.084	0.202	0.384
Adjusted R2	0.089	0.121	0.054	0.171	0.354
R2 change		0.039		0.117	0.182
*F* test for R2 change	4.009 ***	4.277 ***	2.742 **	6.539 ***	12.778 ***

Note: * *p* < 0.05; ** *p* < 0.01; *** *p* < 0.001 (two-tailed).

## Data Availability

Data will be available on request.

## Appendix A. List of Measures

Private-sphere pro-environmental behavior

PRPE1. I print double sided whenever possible

PRPE2. I maintain office equipment

PRPE3. I reuse recyclable materials (e.g., papers/plastic packaging) in the office

PRPE4. I turn lights off when not in use

Public-sphere pro-environmental behavior

PUPE1. I support positive implementation of national and organizational environmental policies

PUPE2. I make suggestions about environmentally friendly practices to managers in an effort to increase my organization’s environmental performance

PUPE3. I remind and persuade colleagues to protect the environment at the workplace

PUPE4. I take part in environmentally friendly programs (e.g., bike/walk to work, bring your own local lunch)

Psychological entitlement

PE1. I honestly feel I’m just more deserving than others in my firm

PE2. I should be treated specially in my firm

PE3. I demand the best because I’m worth it

PE4. I deserve more things in my firm

Rationalization of workplace deviance

RWD1. I generally engage in various types of workplace deviance in order to compete effectively

RWD2. Workplace deviance behavior is a normal part of work

RWD3. The employees who do not engage in workplace deviance will be at a competitive disadvantage compared to those who do engage in these types of actions

RWD4. Workplace deviance behavior is one of the most important considerations when works

RWD5. Workplace deviance is the way to get things well done

Workplace deviance

WD1. He works on his personal matter instead of working for his firm

WD2. He takes an additional or longer break than is acceptable at his workplace

WD3. He often comes in late or leave work early without permission

WD4. He intentionally works slower than he could have worked

WD5. He leaves his work for someone else to finish

WD6. He puts little effort into his work
